# Developing a functional urinary bladder: a neuronal context

**DOI:** 10.3389/fcell.2015.00053

**Published:** 2015-09-01

**Authors:** Janet R. Keast, Casey J. A. Smith-Anttila, Peregrine B. Osborne

**Affiliations:** Department of Anatomy and Neuroscience, University of MelbourneMelbourne, VIC, Australia

**Keywords:** micturition, continence, pelvic ganglion, dorsal root ganglion, inferior hypogastric plexus, visceral afferent, neural development, organogenesis

## Abstract

The development of organs occurs in parallel with the formation of their nerve supply. The innervation of pelvic organs (lower urinary tract, hindgut, and sexual organs) is complex and we know remarkably little about the mechanisms that form these neural pathways. The goal of this short review is to use the urinary bladder as an example to stimulate interest in this question. The bladder requires a healthy mature nervous system to store urine and release it at behaviorally appropriate times. Understanding the mechanisms underlying the construction of these neural circuits is not only relevant to defining the basis of developmental problems but may also suggest strategies to restore connectivity and function following injury or disease in adults. The bladder nerve supply comprises multiple classes of sensory, and parasympathetic or sympathetic autonomic effector (motor) neurons. First, we define the developmental endpoint by describing this circuitry in adult rodents. Next we discuss the innervation of the developing bladder, identifying challenges posed by this area of research. Last we provide examples of genetically modified mice with bladder dysfunction and suggest potential neural contributors to this state.

## Understanding the endpoint: Innervation of the adult urinary bladder

### Overview of bladder innervation

**Figure 1:**
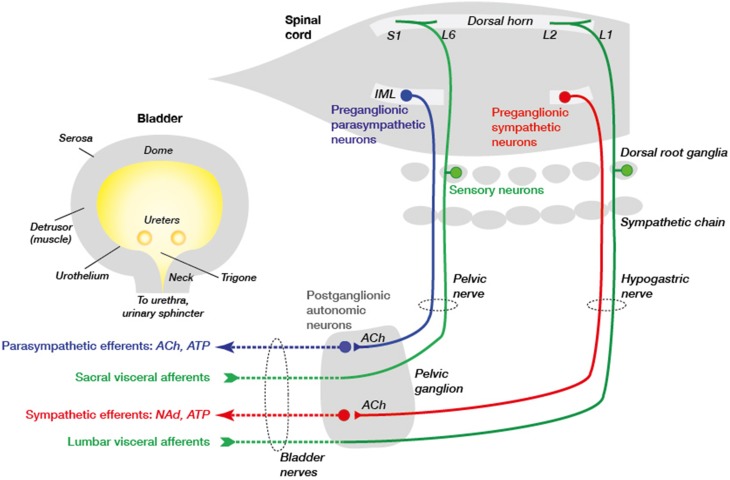
**Primary connections between the spinal cord, ganglia, and urinary bladder**. Sensory neurons (green) in dorsal root ganglia (DRG) lie close to the spinal cord and make connections in the spinal cord and with the urinary bladder tissues. In rodents these DRG neurons are concentrated in spinal levels L1–L2 (“lumbar”) and L6–S1 (“sacral”). They are activated by various physiological and pathophysiological changes within the bladder wall and signal to neurons in the spinal cord and, in turn, the brain. Integrative centers in the brainstem determine whether sympathetic (red) or parasympathetic (blue) pathways are activated to promote, respectively, continence or voiding. These pathways comprise two types of neurons, the first of which are preganglionic neurons in the intermediolateral nucleus (IML) of the spinal cord and the second are postganglionic neurons with cell bodies in the mixed pelvic ganglia. The preganglionic neurons release acetylcholine (ACh) to excite postganglionic neurons in pelvic ganglia that then release different transmitters to tissues in the bladder wall (ACh, NAd [noradrenaline] and ATP). Sympathetic preganglionic neurons also traverse the inferior mesenteric ganglion (not shown) before they reach the pelvic ganglion. Vascular innervation is not shown but is likely to arise from sympathetic chain rather than pelvic ganglion neurons. These autonomic motor pathways are coordinated with sphincter activity, mediated by somatic neurons in the spinal cord (not shown) that directly innervate the striated muscle of the external urethral sphincter. The axons from these neurons are not shown as they do not traverse the pelvic ganglion but project in the pudendal nerve.

The urinary bladder functions to collect and store urine so that it can be released at behaviorally appropriate times. These alternating phases of continence and micturition are precisely controlled by a hierarchical system of neural controls in the spinal cord and brain (Andersson and Wein, 2004; Beckel and Holstege, 2011; de Groat and Yoshimura, 2015). This circuitry connects with the bladder via a system of direct and indirect peripheral neural pathways summarized in Figure 1. In each component of this circuit, nerves involved in bladder control are intermingled with many other nerve types, as discussed in detail below.

This review will provide an overview of the features that govern the organization and function of bladder innervation in laboratory animals and humans. In general, the neural connectivity of the nerve supply to the bladder tissues is similar across species. However, it should be noted that while most contemporary studies use rodents, it was previously more common to use cats, which have larger nerves and are easier to study with neurophysiological techniques. Nevertheless, it is clear that rodents have limitations for studying some specific questions relevant to human clinical urology, such as urethral sphincter control. As species differences cannot be discussed in detail here, the reader is referred to recent reviews that discuss this and other species differences in detail (Andersson and Wein, 2004; Thor and de Groat, 2010; Andersson et al., 2011).

Despite the paucity of data on the development of urogenital innervation and mechanisms of its remodeling in adulthood, the importance of this area of the nervous system is illustrated by the many clinical problems that arise when the nervous system is perturbed. For example, various types of pelvic surgery performed for tumor removal unavoidably damage nearby nerves, resulting in incontinence and sexual dysfunction (Mauroy et al., 2003; Cornu et al., 2014; Wit and Horenblas, 2014; Laterza et al., 2015). Understanding factors that drive growth of these connections during development could identify strategies for their repair in adults. Conversely, chronic cystitis is accompanied by bladder hyperinnervation (Dickson et al., 2006; Boudes et al., 2013) that may contribute to hyperactivity and pain. In this case, identifying growth inhibitory or chemorepulsive factors involved in bladder nerve development may provide targets for reducing aberrant growth and dysfunction in adults.

The nerves of the bladder can be divided into two classes—sensory and autonomic—that differ in their anatomy, chemistry, and function (Jänig, 2008). There are multiple subtypes of sensory and autonomic nerves, as discussed below. As the bladder fills, the bladder wall relaxes to accommodate increasing volume and sympathetic nerves constrict the bladder base and urethra to promote continence. In cats, sympathetic innervation of the detrusor may contribute to continence (Vaughan and Satchell, 1992; Khadra et al., 1995) including by modulating transmission in parasympathetic ganglion neurons (de Groat and Saum, 1972), but this remains a debatable area in rodent research. Continence continues until a threshold tension of the bladder wall is detected by low-threshold mechanosensors (sensory neurons). This can trigger voiding but in healthy adults is dependent on the overriding neural controls from the brain. When suitable to initiate voiding, bladder contraction is activated in coordination with relaxation of the bladder base and urethra, and opening of the external urinary sphincter (striated muscle). This “voluntary” sphincter component of bladder control sets it apart from most other areas of autonomic function (e.g., cardiovascular regulation) that are activated in direct response to changing environmental and bodily needs but are “involuntary” (Jänig, 2008). The innervation of the external sphincter (urethral rhabdosphincter) originates from somatic motor neurons in the spinal cord and will not be discussed further here.

### Sensory innervation of the bladder

Sensory axons form a rich network in most bladder tissues (Gabella, 1995, 1999; Gabella and Davis, 1998; Forrest et al., 2014). They distribute uniformly in the smooth muscle (detrusor) of the bladder wall, but near the urothelium become heavily concentrated at the base and neck—where some axons also penetrate the deeper layers of urothelial cells (Gabella and Davis, 1998; Gillespie et al., 2006; Forrest et al., 2014). This suggests signaling from the urothelium to sensory nerves, which is important in both health and disease (Kanai, 2011; Birder and Andersson, 2013), is spatially restricted. Sensory axons are also found within many blood vessels in the bladder wall (Gabella and Davis, 1998; Forrest et al., 2014) where their role is unknown.

As in other internal organs, sensory axons in the bladder terminate in simple, free endings (Gabella, 1995, 1999; Gabella and Davis, 1998; Forrest et al., 2014). Although often regarded as structurally unspecialized, different branching patterns and terminal structures occur but these variations are relatively subtle in comparison with the large, complex structures at the terminals of specialized sensory nerves in skin and skeletal muscle (e.g., Pacinian and Ruffini corpuscles). These small structural variations have not yet been correlated with particular sensory modalities in the bladder.

Sensory axons in the bladder originate from distant neuronal cell bodies in the lumbosacral dorsal root ganglia (DRG) that lie close to each side of the spinal cord (Figure 1). After leaving the DRG, their axons pass through the pelvic autonomic ganglia *en route* to the bladder, then intermingle with the bladder-projecting motor axons until they reach the bladder wall. Sensory and motor axons cannot be distinguished at this point unless immunolabeled for specific neural markers. As most bladder sensory axons originate from the sacral DRG these neurons are better characterized and more commonly experimentally manipulated than those originating from upper lumbar DRG (Kanai, 2011; Gonzalez et al., 2014; de Groat and Yoshimura, 2015). Given only a minority of neurons in DRG of both spinal levels project to the bladder (Robinson and Gebhart, 2008; de Groat and Yoshimura, 2009), assessing their molecular changes during disease or injury states raises a technical challenge. Anatomical tracing strategies [e.g., retrograde tracer micro-injection into the bladder wall Yoshimura et al., 2003; Forrest et al., 2013; Gonzalez et al., 2014] have been critical for visually separating bladder sensory neurons to define their properties.

Physiological studies recording electrical activity within single axons entering the bladder wall or at their terminals during different types of bladder manipulation (e.g., stretch) have sub-classified them by transduction properties (Zagorodnyuk et al., 2007, 2010; Xu and Gebhart, 2008). The predominant class acts as low-threshold mechanosensors that respond to tension and contraction. However, other classes of low-threshold and high-threshold mechano- and chemo-sensitive nerves can be recruited, especially in response to physical or chemical signals of impending bladder damage (Kanai, 2011; Skryma et al., 2011). These latter signals may be interpreted as sensations of discomfort or pain, but normally the organism does not perceive activity in these neural pathways.

Bladder sensory nerves are either lightly myelinated (A-delta) fibers or unmyelinated (C-fibers) (Kanai, 2011; de Groat and Yoshimura, 2015). Most of the A-delta class comprises low-threshold mechanosensors whereas most C-type fibers are higher threshold sensors that respond to diverse types of stimuli (polymodal), including damage. Measurements of action potential conduction velocity distinguish these two classes, but molecular properties are also useful indicators. For example, the majority of C-fiber bladder sensory neurons express the nociceptive transducer ion channel, TRPV1 (transient receptor potential vanilloid receptor 1), as well as the neuropeptides, calcitonin gene-related peptide and substance P (Arms and Vizzard, 2011; Skryma et al., 2011; Forrest et al., 2013). Functional studies have also used capsaicin-sensitivity to identify activity attributed to bladder sensory neurons that express TRPV1 (de Groat and Yoshimura, 2009; Michel and Igawa, 2015). Conversely, the 200 kD neurofilament protein (NF200) distinguishes the cell bodies of sensory neurons with myelinated axons (Lawson et al., 1993), including the A-delta bladder afferents (Forrest et al., 2013). However, the latter has limited use in the identifying their terminals where neurofilament levels are low (Forrest et al., 2014). Also, NF200 is not diagnostic of sensory axons in the bladder where many autonomic axons express this protein (Forrest et al., 2014).

### Motor innervation of the bladder

The bladder can show myogenic activity that is independently driven by detrusor smooth muscle, but the effectors for the coordinated activity required by continence and micturition are parasympathetic and sympathetic autonomic neurons (Beckel and Holstege, 2011; de Groat and Yoshimura, 2015). Autonomic neurons also control vascular tone in the bladder (Michel and Igawa, 2015).

In the bladder, autonomic axons innervate the same tissues as the sensory axons, albeit in different density—in the detrusor and vasculature, autonomic axons are more prevalent than sensory axons, whereas the converse is true for the urothelium (Gabella and Davis, 1998; Gabella, 1999; Gillespie et al., 2006; Forrest et al., 2014). Although less prevalent than sensory axons, autonomic axons show a similar gradient of urothelial innervation toward the bladder base and neck (Dickson et al., 2006).

Parasympathetic axons release acetylcholine as a transmitter and in some tissues and species release a co-transmitter, ATP (Jänig, 2008). This is the dominant axon type in the detrusor, where they cause muscle contraction during micturition (de Groat and Yoshimura, 2015). Sympathetic axons release noradrenaline (norepinephrine) and potentially also ATP but are scarce in the detrusor, only being found in small numbers in the base and neck of the bladder where they have a role in continence (Kihara and de Groat, 1997; Gosling et al., 1999; Forrest et al., 2014; de Groat and Yoshimura, 2015). A small population of parasympathetic axons lies close to the urothelium, where they are closely associated with sensory terminals (Dickson et al., 2006; Gillespie et al., 2006). The dominant population of sympathetic axons supplies the vasculature (Gabella, 1999; Forrest et al., 2014), where they likely cause constriction.

Most of the autonomic axons innervating the bladder originate from neuron cell bodies in the nearby pelvic ganglia (Figure 1) (Keast and de Groat, 1989). Sympathetic axons innervating the bladder vasculature most likely originate from neurons in the sympathetic chain (paravertebral) ganglia, as is the case for sympathetic vasoconstrictor axons in other organs (Jänig, 2008). The pelvic ganglia of rodents are functionally equivalent to the much more dispersed inferior hypogastric plexus in humans (Keast, 1999; Shoja et al., 2013). Rodent pelvic ganglia comprise paired clusters of thousands of neurons lying close to the uterine cervix and lateral lobes of the prostate, and contain many cell types. First, they contain neurons that each innervate a distinct region of the urogenital tract and lower bowel (Keast and de Groat, 1989; Kepper and Keast, 1995). Therefore, as discussed for identifying bladder sensory neurons in DRG, strategies such as retrograde tracing are valuable. Second, these target-defined neurons comprise both sympathetic and parasympathetic classes (Keast, 1995, 1999; Kanjhan et al., 2003; Jobling and Lim, 2008). Therefore, during development axons from two different regions of the spinal cord converge on the ganglia to synapse with the appropriate type of ganglion neuron. This mixture of sympathetic and parasympathetic neurons in one ganglion occurs nowhere else in the body. In addition to the pelvic ganglia, in rodents a few autonomic ganglion neurons are embedded in the bladder wall (Gillespie et al., 2006; Forrest et al., 2014), albeit are less common than intramural neurons in human bladder (Gilpin et al., 1983).

## Development of bladder innervation

Sensory and autonomic axons innervating the bladder mostly originate from neuronal cell bodies that are some distance from the bladder tissues although some neuronal cell bodies appear transiently in the bladder wall during development and early postnatal life, with very few remaining by adulthood (Zvarova and Vizzard, 2005). Their properties more closely resemble autonomic than sensory neurons (Zvarova and Vizzard, 2005; Forrest et al., 2014) but their function is unknown. Therefore, to understand developmental mechanisms, it is necessary to identifying the relevant neuronal populations within DRG and pelvic ganglia. This includes defining the cues that promote growth and survival, determine guidance and appropriate synaptic targeting, and specify appropriate signaling properties (e.g., ion channel expression, transmitter synthesis). Progress in most of these areas remains limited. Bladder tissues express a typical array of neurotrophic factors (Vizzard, 2000; Vizzard et al., 2000; Gonzalez et al., 2014), and many neurotrophic factor receptors have been identified in neurons within the pelvic ganglia and bladder sensory neurons of the lumbosacral DRG (Murray et al., 2004; Hiltunen et al., 2005; Palma and Keast, 2006; Vizzard, 2006; Forrest et al., 2013; Gonzalez et al., 2014), but their roles in development of bladder innervation have not been defined.

Nerves grow into the bladder tissues well before birth. Axons enter the human fetal urinary bladder by 13 weeks post-conception and their density across each tissue type increases from that time (Kimmel and Mc, 1958; Klück, 1980; Gilpin et al., 1983; Dixon and Jen, 1995; Jen et al., 1995; Dixon et al., 1997, 1998, 1999). In these studies, immunohistochemistry for neuronal markers such as neuropeptides confirms that each of the major chemical classes of axons is likely to be present prior to birth. Moreover, neuronal cell bodies with transmitter chemistry similar to rodent pelvic ganglion neurons are embedded within the bladder wall and on its surface (Gilpin et al., 1983; Dixon et al., 1997); it is likely they are extensions of the inferior hypogastric plexus.

Our understanding of the timing and mechanisms of bladder innervation during embryonic development remains limited. In rodents, neural activation of the detrusor is evident just a few days after birth, but the properties of these contractions (including transmitter and receptor type) continue to change over the first couple of weeks (Levin et al., 1981; Maggi et al., 1984; Kruse and De Groat, 1990; Iuchi et al., 1994; Sann et al., 1997). Numerous images on the GUDMAP database (www.gudmap.org) (Harding et al., 2011) show that in embryonic mice, axons first reach the outer region of the detrusor by embryonic day (E) 14–15, and the urothelium by E18. At this time, axons can be distinguished by immunohistochemical markers that in adults would indicate parasympathetic, sympathetic and sensory axons. However, these may not be reliable markers in developing systems, where gene expression patterns may be quite dynamic. For example, tyrosine hydroxylase identifies adult noradrenergic sympathetic axons, but in development this enzyme is transiently expressed by a much broader neuron population (Rohrer, 2011). There is some early transient expression of cholinergic markers (Rohrer, 2011), and neuropeptide expression may be dynamic. Moreover, if expression of a particular neural marker is absent, it is tempting (but possibly inaccurate) to infer that the axon itself is not yet present.

Bladder-projecting sensory and autonomic neurons comprise only a minority of the neurons in pelvic ganglia and DRG, therefore studying their developmental mechanisms requires their identification (or separation) from surrounding neurons. Whereas, retrograde labeling can be conducted as simple recovery surgery in adult animals, comparable studies cannot be performed in the living embryo. Tracing nerve tracts *in vitro* by applying lipophilic dyes to fixed tissues (Ratcliffe et al., 2006) would be a valuable approach. This may also enable topographic maps to be constructed for developing bladder-projecting neurons, as performed previously for different types of neurons in pelvic ganglia of adult rats (Keast, 1999). In addition to correlating with expression patterns of transmitters, intriguingly expression patterns of transcription factors have been reported in developing mouse pelvic ganglia (Wiese et al., 2012), some of which may correlate with bladder-specific pathways. In parallel with the order of organ maturation, is also possible that bladder-projecting pelvic ganglion neurons differentiate and mature much earlier than those innervating reproductive organs. Therefore, to determine the mechanism by which the bladder becomes innervated, each target tissue within the bladder must be examined, a range of ganglion systems investigated and the neurons within those ganglia that project to bladder identified. Immunohistochemical tools enable distinction of some elements within this system, but without a specific molecular phenotype yet identified for bladder-projecting neurons, progress in defining the route and source of connections will be constrained. Understanding development of mechanisms by which nerves communicate with non-neuronal cells within the bladder, such as glial cells and interstitial cells (Gabella, 1995, 1999; McCloskey, 2011) is also important.

## Genetic models of bladder dysfunction

Animal models provide valuable insights into the factors contributing to particular bladder phenotypes and the mechanisms driving development of normal bladder innervation. Here we highlight some examples of mouse models with dysfunction due to enlarged bladders. We will discuss these in the context of possible neuronal impairment derived from loss of sensory transduction (to detect distension) or motor control (to enable contraction and emptying).

Deletion of the nicotinic acetylcholine receptor Chrna3, or the combined deletion of Chrnb2 and Chrna4, result in a bladder phenotype known as megacystis (Xu et al., 1999a,b). This is characterized by extreme bladder enlargement, overflow incontinence and bladder infection with urinary stones. These features are likely driven by neuronal dysfunction, as nicotinic receptors are expressed by autonomic ganglion neurons and required for transmitting the excitatory message from spinal neurons to ganglion cells, and hence to the organs. Specifically, Chrna3 and Chrnb4 are highly expressed in pelvic ganglia (Park et al., 2006; Girard et al., 2013) and are upregulated in parasympathetic pelvic ganglion neurons in a surgical obstruction model of the urethra (Chung et al., 2015). While not excluding other mechanisms, these reports are consistent with the bladder dysfunction being driven by an inability to void, resulting in distension and hypertrophy of the bladder. This model could provide additional insights into the impact of autonomic inactivity (rather than neuronal loss) on upstream components of the reflex circuitry. The effects of prolonged distension on sensory neurons could reveal mechanisms relevant to a number of clinical obstructive conditions.

A more bladder-specific disruption has been seen with “megabladder” mice (Singh et al., 2007; McHugh, 2014), a phenotype caused by randomly inserting a transgene into chromosome 16 that subsequently, along with a portion of chromosome 16, translocated to chromosome 11 downstream of myocardin. Profound changes, including severe distension and thinning of bladder wall were reported *in utero*, with the bladder almost completely lacking detrusor muscle; less dramatic changes occur in lamina propria and urothelium. This severe problem was restricted to lower urinary tract; muscle development was normal in the gastrointestinal and respiratory tracts, and vascular system. The innervation of these bladder tissues has not yet been examined but could reveal if and how a normal detrusor determines normal bladder innervation patterns. Furthermore, the phenotype was more severe in male, providing a platform to study sex differences in neuronal development.

A different outcome occurred after deleting FGFR2 (fibroblast growth factor receptor 2) from the bladder mesenchyme, leading to thinner detrusor muscle but in this case a thickened, collagen-enriched lamina propria (Walker et al., 2015). This impacted on muscle contractility in response to cholinoceptor and purinoceptor agonists and compliance of the bladder wall. Innervation density was not examined, but their decreased intervoiding interval in cystometry studies provides further encouragement to examine sensory nerve density and threshold for activation in these animals.

## Conclusions

This review highlights the primary features of bladder innervation in the adult in order to demonstrate the gaps to be filled in understanding the development of this anatomically and functionally complex nerve supply. Defining the developmental mechanisms in this system may also reveal strategies to drive regrowth, targeting and functional recovery of bladder nerves in the adult.

### Conflict of interest statement

The authors declare that the research was conducted in the absence of any commercial or financial relationships that could be construed as a potential conflict of interest.

## Abbreviations

Ach: acetylcholine
DRG: dorsal root ganglion
NAd: noradrenaline (norepinephrine)
NF200, neurofilament protein: 200 kd
TRPV1: transient receptor potential vanilloid receptor 1.
